# Olfactory Deficits in Niemann-Pick Type C1 (NPC1) Disease

**DOI:** 10.1371/journal.pone.0082216

**Published:** 2013-12-31

**Authors:** Marina Hovakimyan, Anja Meyer, Jan Lukas, Jiankai Luo, Volker Gudziol, Thomas Hummel, Arndt Rolfs, Andreas Wree, Martin Witt

**Affiliations:** 1 Institute of Anatomy, Rostock University Medical Center, Rostock, Germany; 2 Albrecht-Kossel Institute for Neuroregeneration, Rostock University Medical Center, Rostock, Germany; 3 Department of Otorhinolaryngology, University of Dresden Medical School, Dresden, Germany; Virginia Commonwealth University, United States of America

## Abstract

**Background:**

Niemann-Pick type C disease (NPC) is a rare autosomal recessive lipid storage disease characterized by progressive neurodegeneration. As only a few studies have been conducted on the impact of NPC on sensory systems, we used a mutant mouse model (NPC1^−/−^) to examine the effects of this disorder to morphologically distinct regions of the olfactory system, namely the olfactory epithelium (OE) and olfactory bulb (OB).

**Methodology/Principal findings:**

For structural and functional analysis immunohistochemistry, electron microscopy, western blotting, and electrophysiology have been applied. For histochemistry and western blotting, we used antibodies against a series of neuronal and glia marker proteins, as well as macrophage markers.

NPC1^−/−^ animals present myelin-like lysosomal deposits in virtually all types of cells of the peripheral and central olfactory system. Especially supporting cells of the OE and central glia cells are affected, resulting in pronounced astrocytosis and microgliosis in the OB and other olfactory cortices. Up-regulation of Galectin-3, Cathepsin D and GFAP in the cortical layers of the OB underlines the critical role and location of the OB as a possible entrance gate for noxious substances. Unmyelinated olfactory afferents of the lamina propria seem less affected than ensheathing cells. Supporting the structural findings, electro-olfactometry of the olfactory mucosa suggests that NPC1^−/−^ animals exhibit olfactory and trigeminal deficits.

**Conclusions/Significance:**

Our data demonstrate a pronounced neurodegeneration and glia activation in the olfactory system of NPC1^−/−^, which is accompanied by sensory deficits.

## Introduction

Niemann Pick Type C (NPC) is a fatal autosomal recessive neurovisceral disorder with an estimated prevalence of approximately 1∶150,000 in Western Europe [1]. The disorder is caused by mutations in the NPC1 (in 95% of patients) or NPC2 gene [2].

The NPC1 gene has been identified by positional cloning [3], and its genomic structure was reported two years later [4]. NPC1 gene codes for a membrane protein that contains a sterol-sensing domain and resides in late endosomes [5]. This glycoprotein with a molecular weight of 142 kDa is involved in the intracellular transport of cholesterol, glycolipids and other cellular components.

Mutations in NPC1 lead to a deficient intracellular lipid trafficking, abnormal regulation of cholesterol biosynthesis and intracellular accumulation of unesterified cholesterol and gangliosides GM2 and GM3 in the late endosomes/lysosomes [6], [7]. Clinical symptoms include hepatosplenomegaly, ataxia, dystonia, and progressive neurodegeneration [8], [9]. Most patients die during the first two decades [10].

In feline, canine and mouse animal models of the disease, a similar phenotype is observed involving tremor, ataxia, and other signs of neurologic impairment [11], [12].

The most widely used mutant mouse model of NPC1 disease, named NPC1^−/−^, has a retrotransposon insertion into the N-terminus of the NPC1 gene, along with a 703-bp deletion, causing premature termination of the coding region that excludes most of the sterol-sensing domain [13]. The NPC1^−/−^ mice lack NPC1 protein and exhibit hepatosplenomegaly and progressive neurodegeneration [14]. The symptoms appear at 42–49 days of age with tremors, lack of motor coordination, progressive weight loss, all leading to death by 10 weeks of age [15].

Previous investigations in this NPC1^−/−^ mouse model have reported severe damage and loss of Purkinje cells and other CNS neurons [16], [17], [18] as well as neurodegeneration and transmission defects in the retina [19].

So far, it is not fully understood why defects in NPC1 cause neurodegeneration. Abnormal activity of autophagic/lysosomal systems, which are closely associated with cholesterol accumulation in the endosomal/lysosomal system, has been implicated in NPC1 neuropathology [20], [21]. Microglia- and astrocyte-mediated inflammation has also been proposed to contribute to the progression of neurodegeneration [22]. Except for retinal degeneration [19], sensory systems such as olfactory, trigeminal or auditory pathways in NPC1 disease have not been studied so far.

An important reason to investigate the olfactory system is the unique regenerative nature of some olfactory components. Olfactory receptor neurons can, in contrast to other peripheral neuron-like cells, constantly regenerate from precursor cells. The same is true for central olfactory interneurons that differentiate from neuron precursors migrating from the subventricular zone into the olfactory bulb [23]. Thus, the olfactory system constitutes a prominent example for adult neurogenesis, which may rapidly adapt during neurodegeneration [24].

What is more, many neurodegenerative diseases are associated with early deterioration of olfactory performance. For example, in Parkinson's disease olfactory impairment occurs at least two years before motor symptoms become evident [25]. Similar associations are known for Alzheimer disease [26]–[28], or in the neurologic form of Gaucher's disease, the most common lysosomal storage disorder [29], [30]. In earlier work, we focused on motor acuity and behavioral as well as central molecular aspects in NPC1 [31], [32]. Although the characteristic olfactory impairment in neurodegenerative diseases is well established [33], [34], morphologically distinct regions of the olfactory system have not yet been analysed in NPC1 disease.

Since early diagnosis and a reasonable standard to follow up on disease progression in the patients are crucial for therapeutic intervention in Niemann-Pick type C, we hypothesized a clinical value of olfactory performance in monitoring patients with NPC1 disease. Therefore, in the present study we used NPC1^−/−^ mice to investigate the effects of this disorder on peripheral level, the olfactory epithelium (OE) and the first central relay structure, the olfactory bulb (OB).

## Materials and Methods

### Animals

Heterozygous breeding pairs of NPC1 mice (BALB/cNctr-*Npc1^m1N^*/J, # 3092, The Jackson Laboratories, Bar Harbor, Maine, USA) were used to generate NPC1^−/−^ and control wild type mice. The protocol was approved by the Committee on the Ethics of Animal Experiments of the University of Rostock (approval ID: 7221.3-1.1-088/10) and conducted according to the guidelines for the Care and Use of Laboratory Animals. All efforts were made to minimize suffering.

Mice were maintained on a 12 hours light-dark cycle with water and food ad libitum. The mouse pups were genotyped by using a polymerase chain reaction (PCR) assay.

Nineteen inbred female homozygous NPC1 mutant mice, lacking NPC1 protein (NPC1^−/−^), aged from 5 to 10 weeks, and 16 wild type siblings (NPC1^+/+^) of the same age were used for immunohistochemistry, electron microscopy and PCR/Western blot analysis, and 12 animals of each group were used for the electrophysiological study.

### PCR analysis

For genotyping, 1–2 mm of mice tails were clipped at postnatal day 6 and homogenized in 200 µl DirectPCR-Tail (Peqlab, Erlangen, Germany) supplemented with 20 µl Proteinase K (Qiagen, Hilden, Germany). Three hours of incubation at 56°C and agitation at 1000 rpm on a Thermo Mixer (Eppendorf) were followed by 45 minutes of heating at 85°C to inactivate the proteinase. Samples were then spun at full speed in a benchtop centrifuge for 1 minute. The PCR reactions were performed with 0.5 µl of the obtained extracts. Each lysate underwent two PCRs; Primers 5′-tctcacagccacaagcttcc-3′ and 5′-ctgtagctcatctgccatcg-3′ identified the wild type allele (obtained fragment size 173 bp) and primers 5′-ggtgctggacagccaagta-3′ and 5′-tgagcccaagcataactt-3′ identified the mutant allele (obtained fragment size 475 bp). Both PCRs were carried out under similar cycling conditions, 3′ at 94°C, 3-step cycling 30″ at 94°C - 45″ at 67°C - 45″ at 72°C (35 cycles) and a final elongation for 2′ at 72°C.

### Preparation of the samples

For immunohistochemistry and electron microscopy, the animals were deeply anesthetized with sodium pentobarbital and killed by an overdose of sodium pentobarbital. Then, cardiac perfusion with phosphate-buffered saline (PBS, pH = 7.4) was followed by 4% paraformaldehyde (PFA) in 0.1 M PBS. Subsequently, the heads were cut in median- sagittal direction and fixed by immersion in the same fixative for additional 24 h.

### Antisera and antibodies

Antisera and antibodies used in this study are compiled in Table 1. The polyclonal Galectin-3 (Gal-3) antiserum was kindly provided by Dr. H.-J. Gabius, Munich, and was made and checked for specificity and absence of cross-reactivity to other galectins as previously described [35]. A monoclonal antibody directed against Gal-3, kindly provided by Dr. H. Hughes, London, was checked for specificity and absence of other galectins as described [36].

**Table 1:** Antibodies used in this study, their binding sites in olfactory epithelium and olfactory bulb, and possible functional implications.

Antibody against	MAB/PAB Dilution for IHC/WB	Source, Specification	Pre-treatment	Olfactory epithelium/lamina propria	Olfactory bulb	Function	Reference
Olfactory marker protein (OMP)	rb PAB 1∶4,000/**1∶1,000**	Sigma, Saint Louis, Missouri, USA Cat.#07889 Lot# 017K4829	Micro-waves	Mature ORN	Nerve cell layer, ORN terminals in glomerular layer	Marker for mature ORN, possibly involved in olfactory signal transduction pathway	[72], [73], [74]
Protein gene product 9.5 (PGP 9.5)	rb PAB 1∶2,000	Millipore, Temecula, CA Cat# AB1761 Lot# NG1806826	Micro-waves	Neuronal cells, olfactory neurons	ORN layer, periglomerular cells, tufted cells, mitral cells	Neuron-like properties	[73], [75]
β-III-Tubulin	rb PAB 1∶1,000	Thermo Scientific, Fremont, CA, USA Cat# RB-9249 Lot# KH12448110	Micro-waves	Olfactory neurons	Neuronal cells, olfactory neurons	Neuron-like properties	[76]
MAP-2	MAB MAP2A,2B 1∶1,000 (WB)	Millipore, Temecula, CA, Cat # MAB378 Lot # LV1583055		ORN	Mature neurons	Microtubule assembly, neuron marker	
NeuN	MAB 1∶2,000	Millipore, Temecula, CA Cat# MAB377 Lot# LV1746157	Micro-waves	n/a	All neurons except mitral cells	Marker for neuronal nuclear antigen	[42]
Cathepsin D	rb PAB 1∶2,000**/1∶500**	BioGenex, San Ramon, CA, USA Cat#AR205-5R Lot# PU205-UP	Micro-waves	Macrophages	Microglia	Cell debris degradation	[77]
Galectin-3	rat MAB, undiluted supernatant	Dr. C. Hughes, NIMR, London, UK	Micro-waves	Macrophages	Microglia	Marker for microglia- myelin phagocytosis Adhesion/differentiationInvolved in epithelial differentiation	[35], [36], [44]
	rb PAB, 1∶1,000	Dr. H.J. Gabius, Munich, Germany					
Glial fibrillary acidic protein (GFAP)	rb PAB 1∶2,000/**1∶2,000**	Dako, Hamburg, Germany Cat# Z0334 Lot# 00045904	Micro-waves	Ensheathing cells in the lamina propria	Astrocytes in glomerular layer	Ion balancing; blood-brain barrier	[58], [78]
ABCA-1	rb PAB 1∶1,000**/1∶500**	Novus Europe, Cambridge, UK Cat# NB400-150 Lot# Y-1	Micro-waves	Supporting cells	Neurons and glia cells	Cholesterol efflux pump	[46], [79], [80]
GAPDH	ms MAB 1∶10,000	Abcam, Cambridge, UK, Cat# ab8245				glycolysis	
NPC1	rb PAB 1∶1,000	Abcam, Cambridge, UK, Cat# 106534		ORN, ensheathing cells, fibroblasts, endothelial cells	ORN, ensheathing cells, astrocytes, endothelial cells	Cholesterol trafficking	[31]

### Immunohistochemistry

The specimens were decalcified in 1% EDTA for 6–24 h at room temperature (RT) or 37°C, shortly rinsed in distilled water, dehydrated and embedded in paraffin. Heads were cut in the sagittal plane in order to visualize both OE and OB on the same section (thickness 5 µm). In order to improve antigen retrieval, deparaffinized and re-hydrated tissue sections were pretreated with microwaves [37] (10 min. in 0.05 Mol/l citrate buffer, pH 6, 800 W) (see Table 1) and exposed to 0.3% aqueous H_2_O_2_ to block endogenous peroxidases. The sections were then incubated with various primary antibodies as listed in Table 1 (pH 7.2; containing 1% bovine serum albumin) for 1 h at 37°C. After washing in PBS, the sections were exposed to biotinylated secondary antibodies for 45 min. at RT. The reaction products were visualized by an avidin-biotin-peroxidase complex (ABC; Vectastain-Elite; Vector, Burlingame, CA, USA) followed by incubation with 0.3% diaminobenzidine/H_2_O_2_ according to the ABC technique [38]. Sections were counterstained with hematoxylin.

### Indirect immunofluorescence

For co-localization experiments a double immunofluorescence protocol was performed as described earlier [39]. Briefly, paraffin sections were dewaxed and incubated with antisera against GFAP followed by donkey anti rabbit Texas Red secondary antibody (1∶80; Molecular Probes, MobiTec, Göttingen, Germany) at 37°C for 1 h. Subsequently, the sections were incubated with either the microglia marker anti-Gal-3 or anti- olfactory marker protein (OMP) at 37°C for 1 h followed by incubation with donkey anti-goat or donkey anti-rabbit FITC secondary antibody (1∶80) for 30 min at 37°C. The sections were mounted in buffered glycerine gelatine and observed with an Olympus BX60 microscope. Photographs were taken using a CCD camera connected to a soft-imaging analysis system (Olympus ANAlysis, Münster, Germany). Separate images for GFAP and Gal-3/OMP immunohistochemistry were obtained from double-labelled specimens, and the individual images were colour-separated into their RGB components. The red (GFAP) and green (Gal-3, OMP) were merged and the composite images imported as TIFF files into Adobe Photoshop CS2 (Adobe Systems) for size reduction.

The following controls were carried out: (1) omission of the primary antibody to rule out non-specific binding of the secondary antibodies and (2) parallel incubation of tissue previously reported to be immunoreactive to the markers tested.

### Electron microscopy

After initial perfusion and preparation (see above), samples of five NPC1^−/−^ and NPC1^+/+^ mice were postfixed in 0.1M cacodylate buffer containing 2.5% glutaraldehyde for at least 24 hours at 4°C. Subsequently, turbinates containing olfactory mucosa and cross-sectioned samples of the olfactory bulb (OB) as well as trigeminal ganglia were excised and kept in the same fixative. Thereafter, the specimens were osmicated, washed, block contrasted with 2% aqueous uranyl acetate, dehydrated through a graded series of ethanol, and embedded in Epon 812 (Plano GmbH, Marburg, Germany). Ultrathin sections (about 70 nm) were mounted on pioloform-coated slot copper grids and contrasted with uranyl acetate (4 minutes) followed by lead citrate (2 minutes). The specimens were examined with a Zeiss EM 902 transmission electron microscope (Zeiss, Oberkochen, Germany) at 80 kV. Photographs were taken using a CCD camera (Proscan, Lagerlechfeld, Germany) and adjusted using Photoshop CS2 software (Adobe Systems).

### Western blot analysis

Animals subjected to western blotting were sacrificed by cervical dislocation. Tissue lysates were obtained by the addition of ice-cold RIPA buffer (500 µl/100 mg tissue) and passing through a 1 ml syringe equipped with a 20×g needle. Subsequent sonication with a UP200S Ultrasonic Processor (Hielscher, Teltow, Germany) (2×15 sec. on/off pulses with a two minute cooling on ice in between) was applied to ensure that homogenisation was completed. Typically, 50 µg of lysate were loaded on a 4–15% Criterion™ precast polyacrylamide gel (Bio-Rad, Munich, Germany). Semiquantitative Western blot detection was carried out using the Odyssey Infrared ImagingSystem (LI-COR Biosciences GmbH, Bad Homburg, Germany) according to protocols described previously [40]. Antibodies (for sources see Table 1) against the following proteins were used: Cathepsin D (1∶500), beta-III tubulin (1∶1,000), ABCA-1 (1∶500), NPC1 (1∶1,000), MAP2 (1∶1,000), OMP (1∶1,000), GFAP (1∶2,000), and GAPDH (1∶10,000), which served as a loading control. Alexa-Fluor 680 and goat anti-mouse IRDye 800 (1∶10,000; Invitrogen) were used as secondary antibodies.

### Electro-olfactometry

#### Animal preparation for EOG recording

The recordings were performed as described earlier [41]. Twenty-four mice [divided in two groups of 31 day-old and 67-day-old animals, respectively] were investigated. Mice were killed by cervical dislocation. Heads were cut median-sagitally. The nasal septum and the mucosa of the nasal wall were dissected under a stereo microscope. The nasal mucosa was kept moist with Ringer's solution and stored at 4°C.

#### Olfactometer and olfactory stimuli

Volatiles were applied using a calibrated air-dilution olfactometer (OM2sl; Burghart Instruments, Wedel, Germany) that allows embedding the stimuli in a constant air flow (2 l/min) of constant temperature (36.5°C) and humidity (80% relative humidity). Phenylethyl alcohol (PEA) (40% and 20%, v/v) and hydrogen sulfide (H_2_S) (40% and 20%, v/v) were used as olfactory stimuli, and CO_2_ (50% and 40%, v/v) was used as trigeminal stimulus. Control stimulation was performed using odorless air.

#### EOG recording

A powerlab 26T device (AD instruments, Bella Vista, Australia) and Chart 5.5.5 for WindowsTM were used to record the EOG. Recordings from the olfactory mucosa were made using tubular electrodes made from TeflonTM tubing (Labokron, Sinsheim, Germany; outer diameter 0.8 mm), filled with 1% Ringer-agar (Agar: Sigma-Aldrich Chemie GmbH, Steinheim, Germany) and containing a silver chloride coated silver wire (electrode resistance <5 kOhm).

## Results

### Animals

Adult NPC1^−/−^ mice (65–70 d) exhibited a reduced body weight compared to control litter mates (17.6 g±2 g in NPC1^−/−^ vs. 20.7 g±1.5 g in NPC1^+/+^, p = 0.0221). Young animals (30 d, typically showing first neuropathology symptoms and cellular alterations, see below) did not yet show significant weight differences (13.4 g±2.4 g in NPC1^−/−^ vs. 15.9 g±2.6 g in NPC1^+/+^, p = 0.1014). Brain weights of old animals were significantly decreased (0.38 g±0.01 g in NPC1^−/−^ vs. 0.43 g±0.02 g in NPC1^+/+^, p = 0.0221, Fig. S1).

### Immunohistochemistry

#### Structural features of olfactory epithelium and olfactory bulb

**Figure 1:**
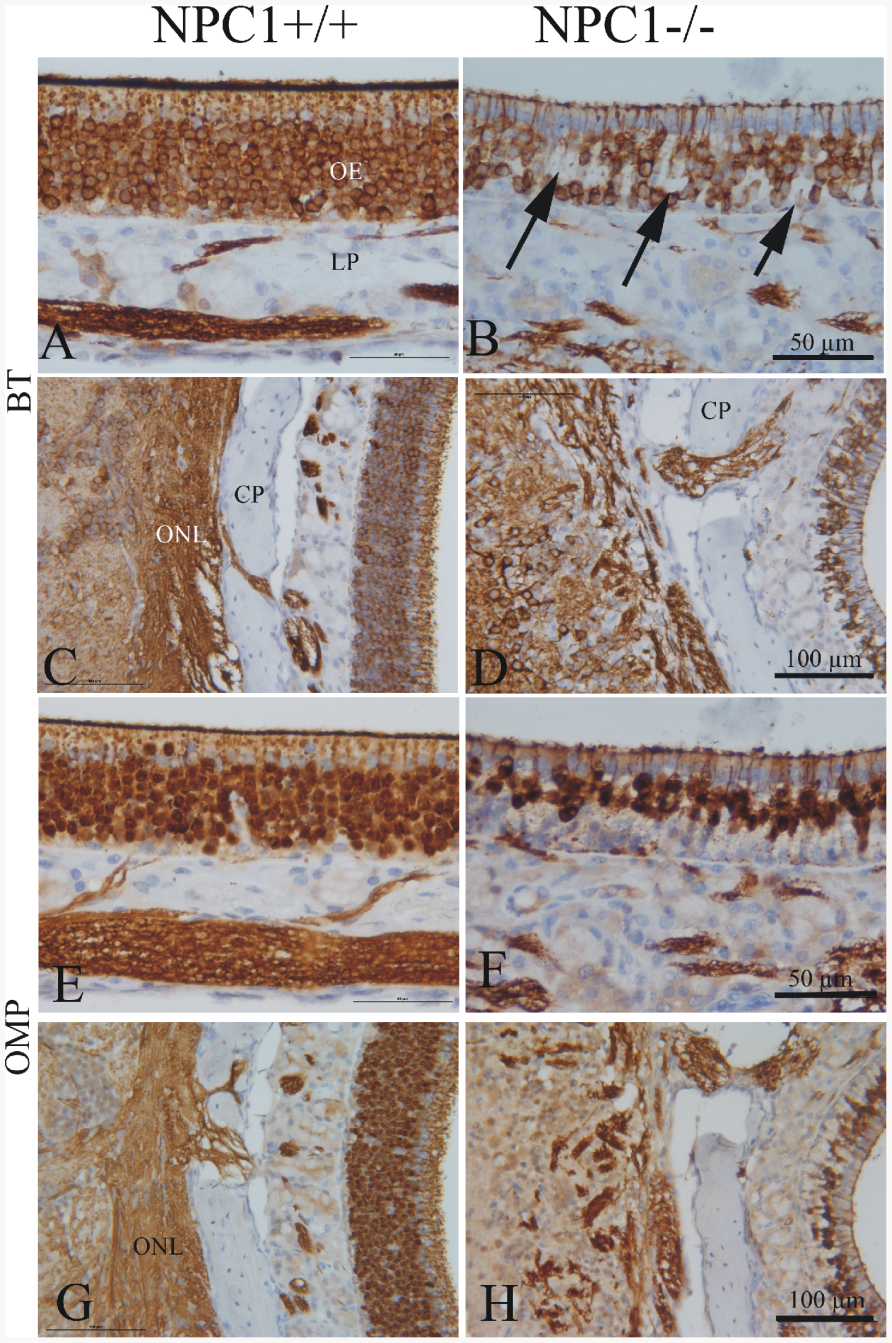
Immunohistochemistry of olfactory epithelium (OE) and olfactory bulb (OB) in adult NPC1^+/+^ and NPC1^−/−^ mice (67–70 d). Beta-tubulin (BT, A–D); Olfactory marker protein (OMP, E–H). (A) Regular columnar epithelium (OE) and olfactory nerve fibers in the lamina propria (LP). In contrast, the number of BT-positive cells is markedly decreased (B). There are large gap-like spaces in the suprabasal area of the epithelium (arrows). (C) Olfactory mucosa (right side) and nerve fiber layer of the OB show a normal morphology. Bundles of olfactory nerve fibers travel through gaps of the cribriform plate (CP) into the olfactory nerve layer (ONL) of the OB (left side). (D) ORN are more scattered and appear interrupted in the NPC1^−/−^ animal. (E, G) OMP reactivity in NPC1^+/+^, detected in mature ORN, is comparable to the distribution of BT (see A and C). NPC1^−/−^ mice (F, H) exhibit similar deficits as shown for BT in B and D. OMP antigen is also located in nuclei. Counterstained with hematoxylin.

**Figure 2:**
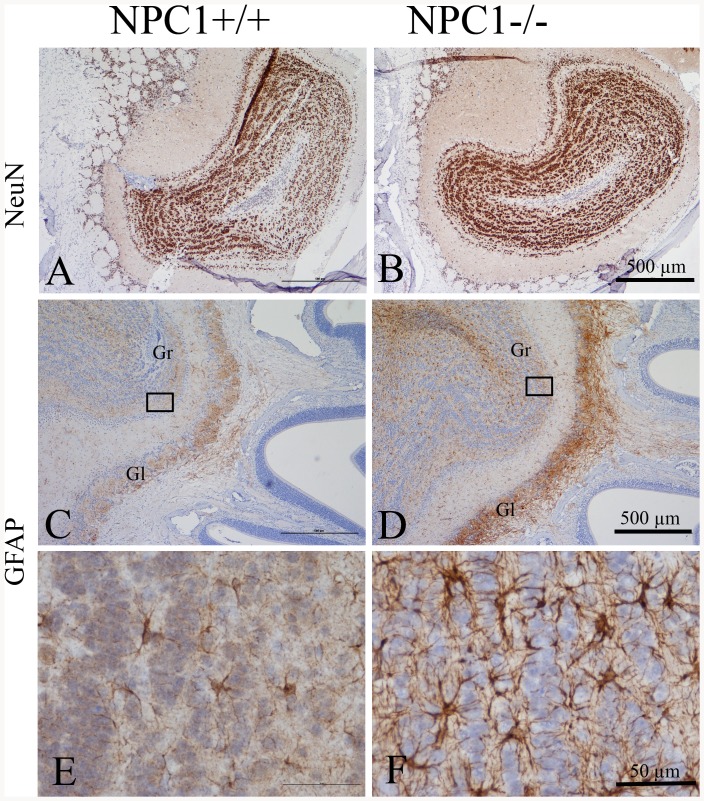
Immunohistochemistry of NeuN (A, B) and GFAP (C through F) in the OB. Distribution of NeuN-positive neuronal cells do not reveal any obvious differences between NPC1^+/+^ (A) and NPC1^−/−^ mice (B) (67–70 d). There is a clear increase of GFAP expression, especially in the glomerular layer (Gl) in NPC1^−/−^ animals (D) compared to NPC1^+/+^ (C). The area marked by the rectangles is enlarged in E and F, respectively, demonstrating the astrogliosis of the granular layer (Gr) in NPC1^−/−^ mice. Counterstained with hematoxylin.

**Figure 3:**
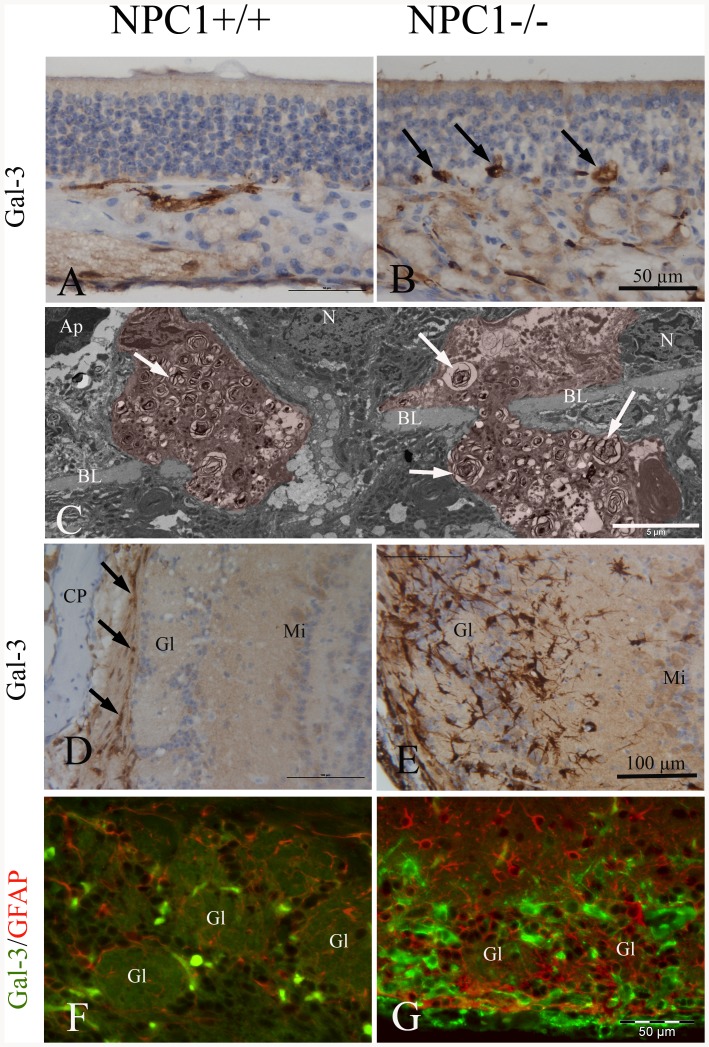
Immunohistochemistry of Gal-3 in the OE (A, B) and OB (D, E) (67–70 d). (A) Gal-3 in NPC1^+/+^ is restricted to olfactory axon bundles in the lamina propria, whereas in NPC1^−/−^ animals (B), large spot-like Gal-3 reaction product is detectable above and below the basal membrane. Note tender processes of these cells, presumably macrophages (arrows). These cells correspond most likely to those depicted in C. (C) Interface between olfactory mucosa and lamina propria. Electron microscopic resolution of two macrophages (red) filled with myelin-like material (arrows). These cells penetrate the basal lamina (BL). N- nuclei of horizontal basal cells; Ap- nucleus of an apoptotic cell. (D) NPC1^+/+^, cortical layers of the olfactory bulb express Gal-3 immunoreactivity only in nuclei of ensheathing cells within the nerve fiber layer (arrows). Deeper bulb areas, i.e., as glomerular (Gl), mitral cell (Mi), and granular (Gr) layers, are not affected. (E) Numerous Gal-3-positive cells occupy the glomerular and adjacent part of the external plexiform layer in an NPC1^−/−^ animal. These cells have partly short and interrupted processes different from those of astroglia. Glomeruli are barely recognizable. (F, G) Double immunofluorescence, using antibodies against GFAP (red) and Gal-3 (green). (F) GFAP reactive astrocytes are mainly distributed within the glomerular layer. There are only a few Gal-3-positive microglia cells in NPC1^+/+^ animals. (G) Prominent microgliosis (Gal-3 expression) especially around glomeruli goes along with enhanced reactivity for GFAP, indicating which indicates astrogliosis. Macrophage activity is most likely associated with microglia rather than with activated astrocytes. Astrocytes do not co-express Gal-3.

**Figure 4:**
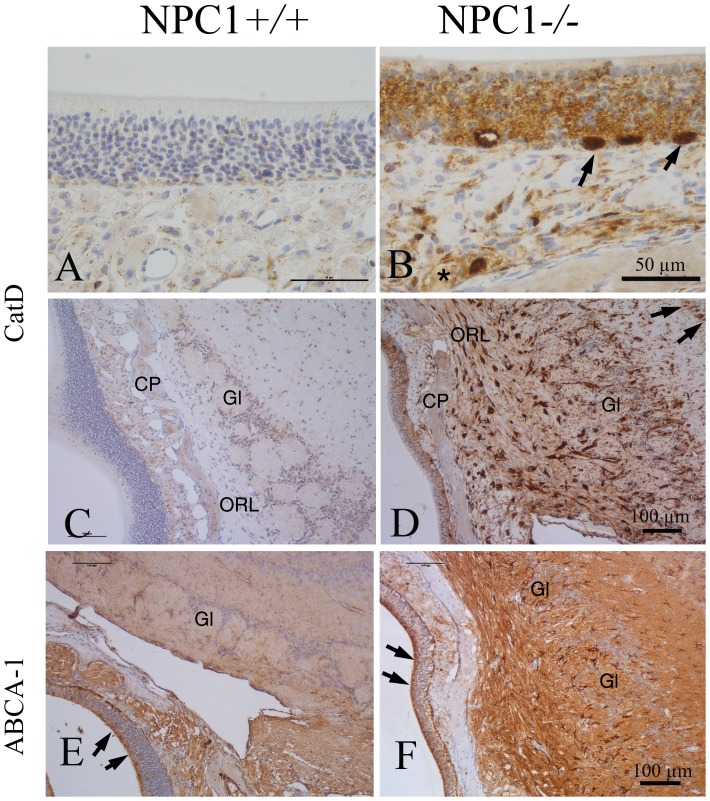
CatD (A–D) and ABCA-1 (E–H) immunoreactivity. (A) Normal distribution of CatD reactivity in NPC1^+/+^; typical dot-like residues in cells of the lamina propria and basal cells of the olfactory epithelium. (B) Dramatically increased CatD reactivity is detected in all epithelial cells in NPC1^−/−^, especially suprabasal cells, most probably macrophages as demonstrated in Figs. 4C and 8. Also enhanced reactivity also of ensheathing cells of ORN bundles in the lamina propria (asterisk). (C) Overview of the interface between OE and OB in NPC1^+/+^ mice. (D) CatD reactivity is dramatically increased in all layers of the OB, including mitral cells (small arrows, upper right corner) and periglomerular cells in NPC1^−/−^. Gl, glomerular layer, CP, cribriform plate. (E, F) Reactivity of ABCA-1 is increased in nerve fibers of NPC1^−/−^ animals (F), whereas supporting cells (arrows) of the OE displays similar intensities in both wild type (E) and mutant (F) animals.

Light microscopic analysis of the olfactory epithelium (OE) of NPC1^+/+^ and NPC1^−/−^ mice revealed obvious differences between groups (Figs. 1–4).

In general, the NPC1^−/−^ group displayed highly disorganized OE. The pan-neuronal marker beta-III-tubulin (BT) immunoreactivity demonstrated a highly organized columnar appearance of olfactory receptor neurons (ORN) in the OE of NPC1^+/+^ mice (Fig. 1A). In contrast, the architecture of ORN was severely disturbed in OE of NPC1^−/−^ mice, showing apparently enlarged gap-like spaces in the basal and middle portions of the epithelium (Fig. 1B).

In the OB the most obvious differences between two phenotypes occurred in the olfactory nerve layer (ONL, Fig. 1C and D). While fibers of the ONL of NPC1^+/+^ mice strongly reacted with BT antibody and revealed a continuous appearance (Fig. 1C), their continuity was severely disturbed, and BT immunoreactivity appeared reduced in OB of NPC1^−/−^ mice (Fig. 1D).

Olfactory marker protein (OMP), an established marker for mature olfactory cells, showed a dark and homogeneous reaction pattern in the OE of NPC1^+/+^ mice (Fig. 1E). OMP immunostaining was appreciably less dense in the OE of NPC1^−/−^ mice (Fig. 1F). Particularly, the basal third of the OE revealed no detectable OMP reactivity. Also the ONL of the OB displayed a much more disrupted organization of OMP-staining in NPC1^−/−^ mice (Fig. 1H) when compared to NPC1^+/+^ (Fig. 1G).

#### Neuronal and astroglial phenotype in OB of NPC1^+/+^ and NPC1^−/−^ mice

The immunolabeling for NeuN, a neuronal nuclear antigen [42], showed a strong reactivity in granule cells in OBs of NPC1^+/+^ and NPC1^−/−^ mice (Fig. 2A and B, respectively). Interestingly, the granule cells in NPC1^−/−^ mice OB demonstrated relative normal density and distribution, comparable to controls. The largest cells, mitral cells, are not recognized by NeuN antibodies.

In NPC1^−/−^ mice, the most intense reactivity of GFAP antibody was attributed to astrocytes within the glomerular layer of the OB (Fig. 2D). Compared to the few astrocytes with short processes in NPC1^+/+^ mice (Fig. 2C, E), here, abundant GFAP-positive astrocytes with their processes spread out in several directions were present (Fig. 2F). The external plexiform layer seemed not to be affected, and the density and distribution of GFAP-immunoreactive cells was comparable to that in NPC1^+/+^ mice.

#### Galectin-3 expression as a marker for inflammatory processes and microglia activation

To explore the inflammatory processes, immunohistochemistry for Galectin-3 (Gal-3) was performed (Fig. 3). Gal-3 is highly expressed and secreted by peripheral macrophages and microglia [43], [44] and is associated with chronic inflammatory and fibrotic processes [45]. While Gal-3 expression was negligible in OE of NPC1^+/+^ mice (Fig. 3A), the number of Gal-3-positive cells was clearly increased in OE of NPC1^−/−^ mice (Fig. 3B). Here, cells strongly positive for Gal-3 were visualized, particularly near the basal membrane (Fig. 3B). Additionally, the presence of macrophages in the basal epithelial layer was identified by electron microscopy. Figure 3C demonstrates macrophages crossing the basal membrane filled with myelin-like laden autophagosomes.

Similarly to the OE, also the OB of NPC1^−/−^ mice exhibited high numbers of Gal-3-immunoreactive cells (Fig. 3E) when compared to NPC1^+/+^ animals (Fig. 3D). Particularly, accumulation of these cells was seen in the glomerular cell layer (Fig. 3E).

To clarify if the excessive Gal-3 production was associated with activated astrocytes, a double immunofluorescence reaction was performed against Gal-3 and the astroglial marker GFAP (Fig. 3F, G). In good agreement with light microscopy, only a few scattered Gal-3 positive cells were identified in the NPC1^+/+^ OB (Fig. 3F), while the OB of NPC1^−/−^ mice showed a clearly increased number of Gal-3 positive cells (Fig. 3G). However, no co-localization of Gal-3 and GFAP could be observed suggesting that macrophage activity was most likely associated with microglia rather than with activated astrocytes.

#### Cathepsin D expression level for evaluation of lysosomal activity

Cathepsin D (CatD) was used as a marker enzyme to evaluate the endolysosome function in OE and OB of both groups. A faint background staining was seen in all cellular layers of OE in NPC1^+/+^ group (Fig. 4A, C). In contrast, large (20–30 µm) CatD immunoreactive cells, resembling macrophage-like cells as already shown in Fig. 3B, were seen in the basal epithelium of NPC1^−/−^ mice (Fig. 4B). Furthermore, CatD-positive cells were identified in the lamina propria. Also an appreciable immunoreactivity for CatD became obvious in the OB of NPC1^−/−^ mice (Fig. 4D). These cells were distributed throughout the entire OB, being most abundant in ONL and glomeruli. When compared to NPC1^−/−^, the OB of the NPC1^+/+^ group exhibited only a few occasional positive cells (Fig. 4C).

#### ABCA-1 expression as a marker for lipid efflux

The ATP-binding cassette transporter 1 (ABCA-1) constitutes the major mediator of cellular cholesterol across the plasma membrane [46]. ABCA-1 is expressed mainly by sustentacular cells of the OE and by numerous cell processes within the OB (Fig. 4E). Compared to NPC1^+/+^ individuals, the overall expression of ABCA-1 in NPC1^−/−^ mice was clearly increased throughout the OB (Fig. 4F).

#### Age-related differences

In comparison to adult (65–70 d) NPC1^−/−^ mice, young animals (35 d) generally showed less distinct, though already clearly visible abnormalities with respect to all markers used in this study (data now shown, except for electron microscopy and electrophysiology, see below).

### Western blot analysis

**Figure 5:**
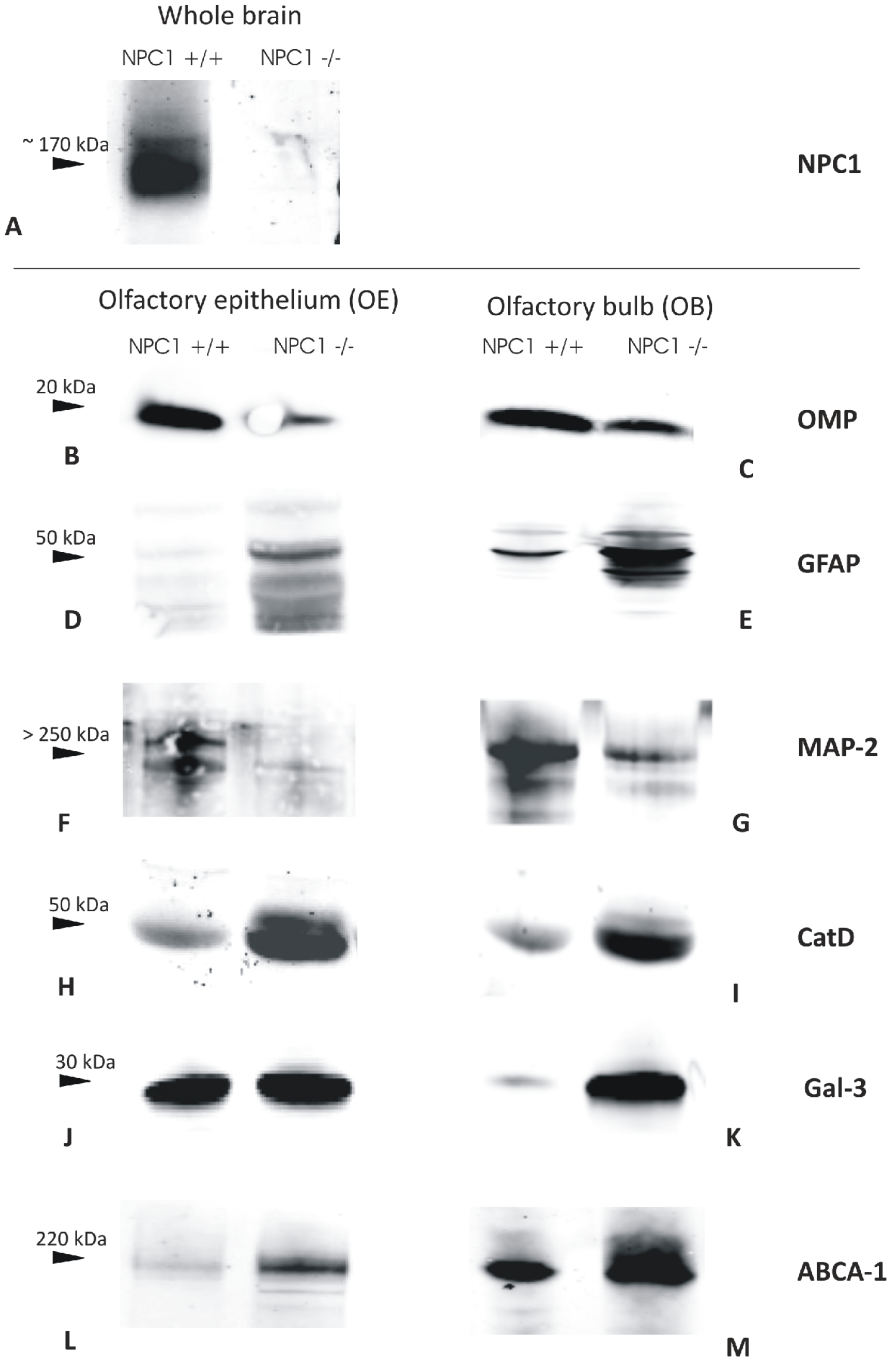
Western blot analysis of the olfactory system in NPC1^−/−^ mice compared to normal controls. **A**: NPC1 deficiency of the mice in the brain. **B/C**: OMP is reduced in OE and OB. **D/E**: The signal for gliosis marker GFAP is enhanced in the NPC1^−/−^ mouse in both OE and OB. **F/G**: Marker of mature neurons MAP-2 shows a decreased signal in OE and OB. Pro-inflammatory and macrophage markers CatD (**I**) and Gal-3 (**K**) were elevated in OB. For CatD, a pronounced increase was observed also in OE (H). **L/M**: ABCA-1, a transporter known to respond to intracellular cholesterol dysregulation is up-regulated in the OE and OB of NPC1 deficient animals.

To confirm the immunohistochemical findings, respective protein levels were examined using standard immunoblotting techniques (Fig. 5). Age-matched (>P60) NPC1^−/−^ (end-stage of the disease) and wild type control animals were subjected to this analysis. Firstly, it was confirmed that the mice were deficient of NPC1 protein. Herein, whole brain lysates served as specimen (Fig. 5A). Qualitative analysis of the blots revealed a lower expression level of neuronal marker OMP in the OE and OB of NPC1^−/−^ mice (Fig. 5B and C, respectively). In good agreement with the gliosis observed in immunohistochemistry in the NPC1^−/−^ group, the levels of astroglial marker GFAP were increased both in OE (Fig. 5D) and OB (Fig. 5E). An apparent neuronal decline was evidenced by a remarkable decrease in neuronal MAP-2 protein in the NPC1^−/−^ group (Fig. 5F/G).

Also the NPC1^−/−^ group demonstrated elevated CatD levels when compared to NPC1^+/+^ control mice, and this increase was seen both, in OE (Fig. 5H) and OB (Fig. 5I). Compared to the control group, a distinct increase in Gal-3-expression became obvious in OB (Fig. 5K), rather than in OE (Fig. 5J) of NPC1^−/−^ mice.

Increased expression of ABCA-1 was noted in the OE (Fig. 5L) as well as in the OB (Fig. 5M) in the NPC1^−/−^ group.

### Transmission electron microscopy

**Figure 6:**
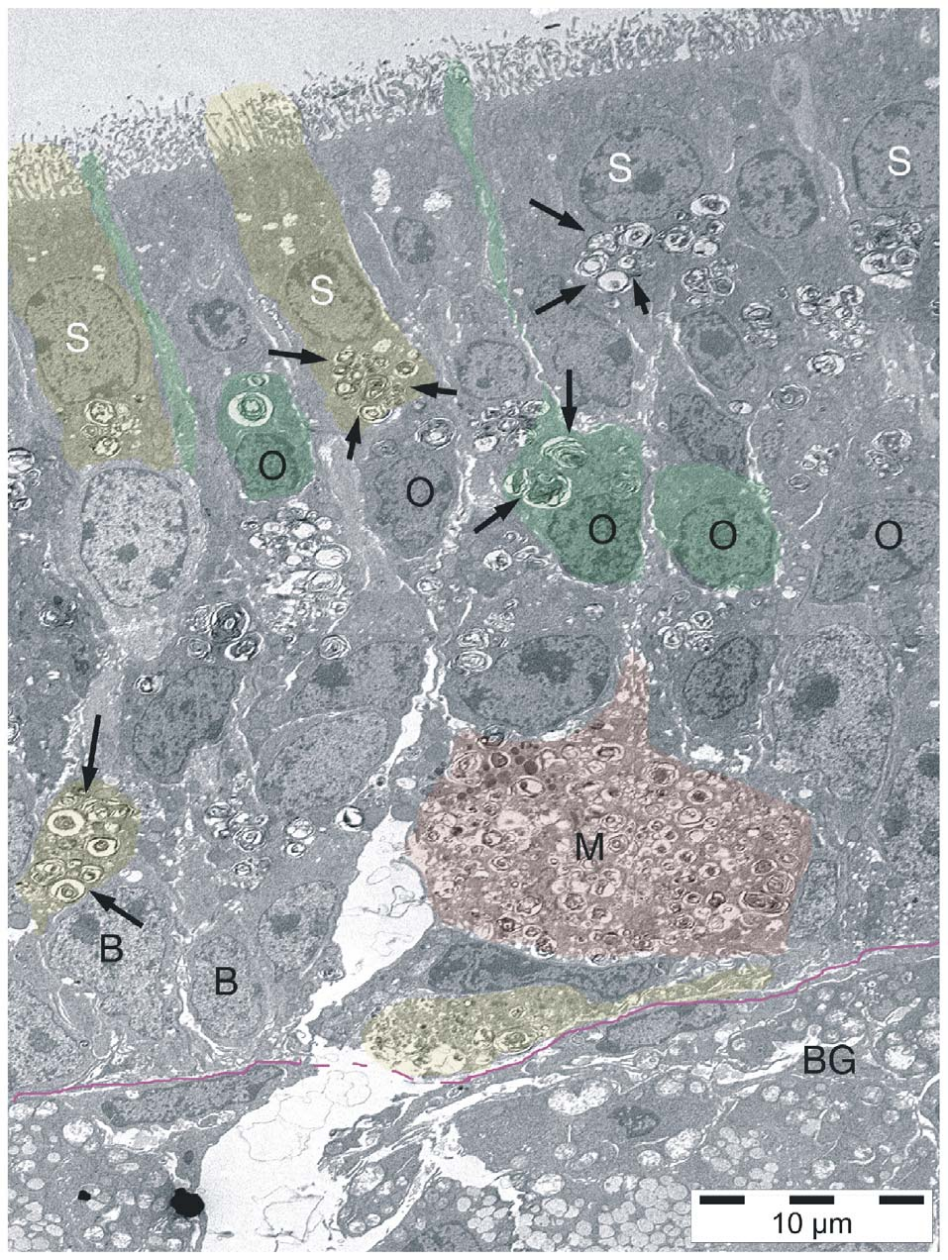
Electron microscopic depiction of the olfactory epithelium of a young (32 d) NPC1^−/−^ animal. Some of the cells are exemplarily colored. The OE consists 1) of olfactory receptor neurons in the middle portion of the OE (O, green) with thin apical processes extending with knob-like structures into the mucous layer, 2) supporting cells, the nuclei of which lie in the upper third, and with basal footplates (S, yellow) above the basal membrane (magenta line), and 3) basal cells (B). There are many irregular, myelin-like inclusions within most of the cells (arrows). Most of these autophagosomes occur in subnuclear portions of supporting cells, but also, to a lesser degree, in perinuclear locations of ORN. Apical processes of ORN are not affected (see also Fig. 7). Singular, huge accumulations of myelin-like deposits are occasionally seen near the basal membrane, most likely corresponding to Gal-3 – and CatD-positive macrophages (M, red; see also Figs. 4,5). BG, Bowman glands of the lamina propria.

**Figure 7:**
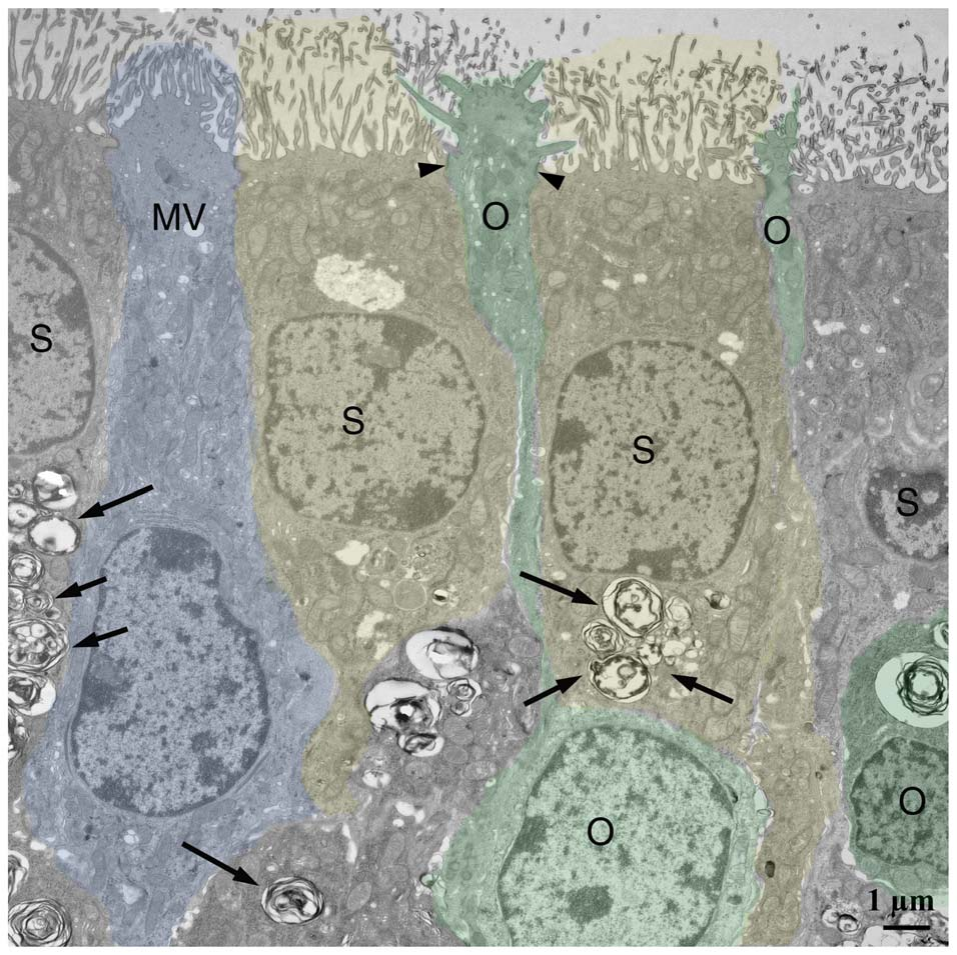
Superficial part of the olfactory epithelium in an NPC1^−/−^ animal. Most prominent are perinuclear autophagosomes in supporting cells (yellow, arrows). ORN (green) occasionally contain such deposits, but appear normal at the surface. Tight junctions are intact (arrowheads). One microvillar cell (MV) is not affected.

**Figure 8:**
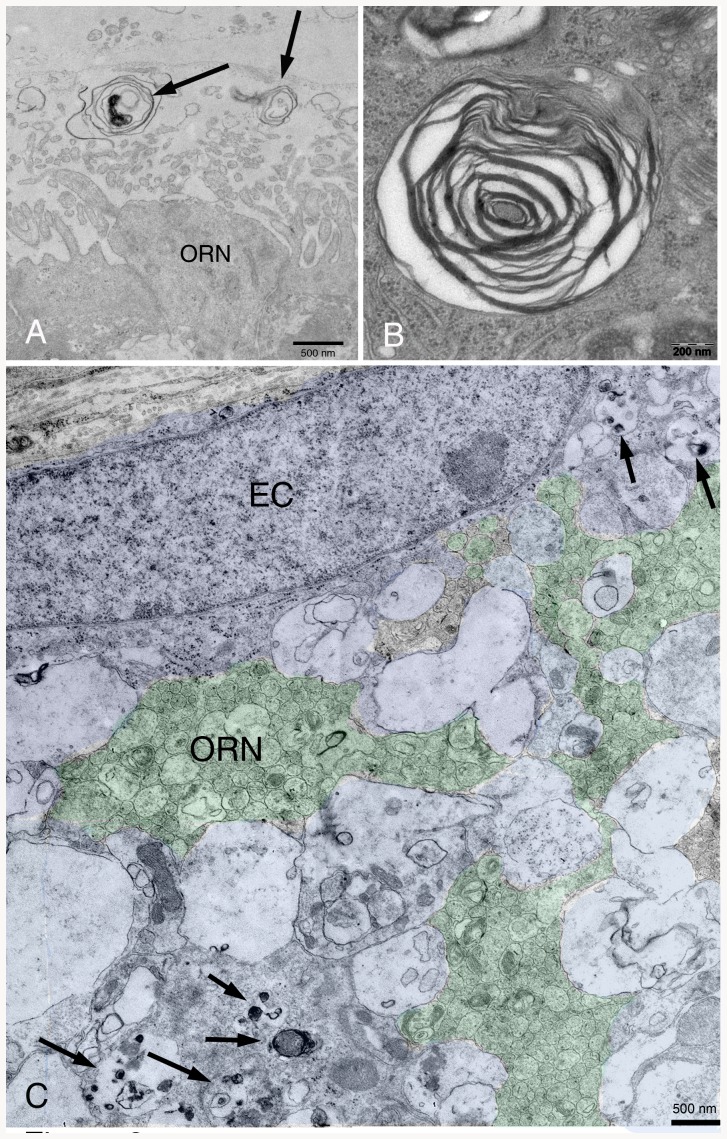
(A) Apical surface of the OE in an NPC1^−/−^ animal. Myelin-like figures (arrows) seem to be shed from one of the OE epithelial cells into the mucous layer. (B) Characteristic pattern of multilamellar deposits within one of the ORN in the middle of the OE. (C) Lamina propria with an ensheathing cell (EC, blue), enwrapping axons of ORN (green). Membrane fragments and vesicular deposits are accumulated exclusively in dilated cisterns of EC, but not in ORN.

**Figure 9:**
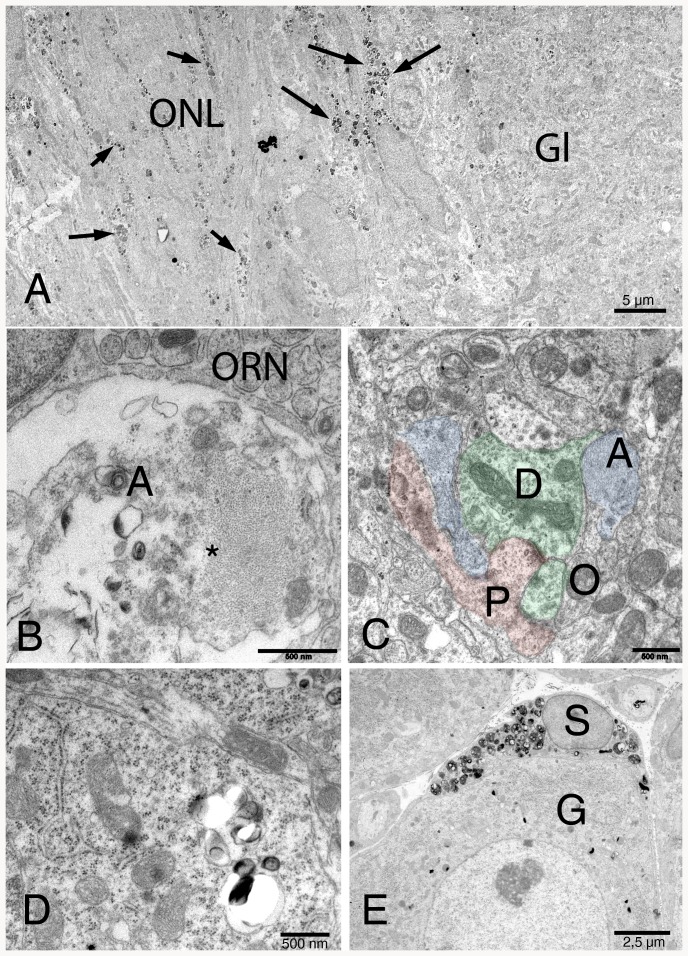
(A) Low power resolution of the olfactory nerve fiber layer (ONL, right) and a glomerulus (GL, left). Similar to the situation in the lamina propria, numerous autophagosomes are seen in ensheathing cells (arrows). The glomerulus is almost free of these deposits. (B) High power resolution of a large degenerating astrocyte cell process (A) with remnants of membranes, vesicles and intermediate filaments (asterisk). Olfactory receptor neurons (ORN) are intact. (C) Dendritic compartment of a glomerulus. Synaptic connectivity seems not to be disrupted, and cells are largely free of deposits. *D*- mitral cell dendrite (green); *P*, periglomerular cell (red), *A*, astrocyte (blue); *O*, axon of olfactory receptor neuron. (D) Perikaryon of a mitral cell with autophagosomes containing membranous material. (E) Trigeminal ganglion cell (G) and an enwrapping satellite cell (S). The latter is packed with autophagosomes.

NPC1^−/−^ animals present myelin-like lysosomal deposits in virtually all types of cells of the peripheral and central olfactory system of NPC1^−/−^ mice (Figs. 6–9). Supporting cells of the OE upper third (Fig. 7), olfactory ensheathing cells of the lamina propria (Fig. 8) and central glia cells (Fig. 9A–D) were especially affected resulting in astrocytosis and microgliosis in the olfactory bulb. Unmyelinated olfactory afferents of the lamina propria seem less affected than ensheathing cells. Figure 7 demonstrates typical myelin-like lysosomal inclusions deposited in autophagosomes in supporting cells and ORN perikarya of the OE. Some myelin figures seem to be shed into the mucous layer of the mucosa (Fig. 8A). Olfactory ensheathing cells exhibit enlarged vacuole-like compartments of ER and autophagosomes (Fig. 8C). The number of autophagosomes is especially high in ensheathing cells of the nerve fiber layer of the OB (Fig. 9A), but also astrocytes (Fig. 9B) and mitral cells (Fig. 9C) and their dendrites are affected. Massive accumulation of myelin-like material was observed in microglia close to capillaries and endothelial cells (not shown). Comparisons between young (32 d) and adult (67 d) animals show that deposits in OE of young animals is already abundantly present (Fig. S2).

### Electrophysiology

**Figure 10:**
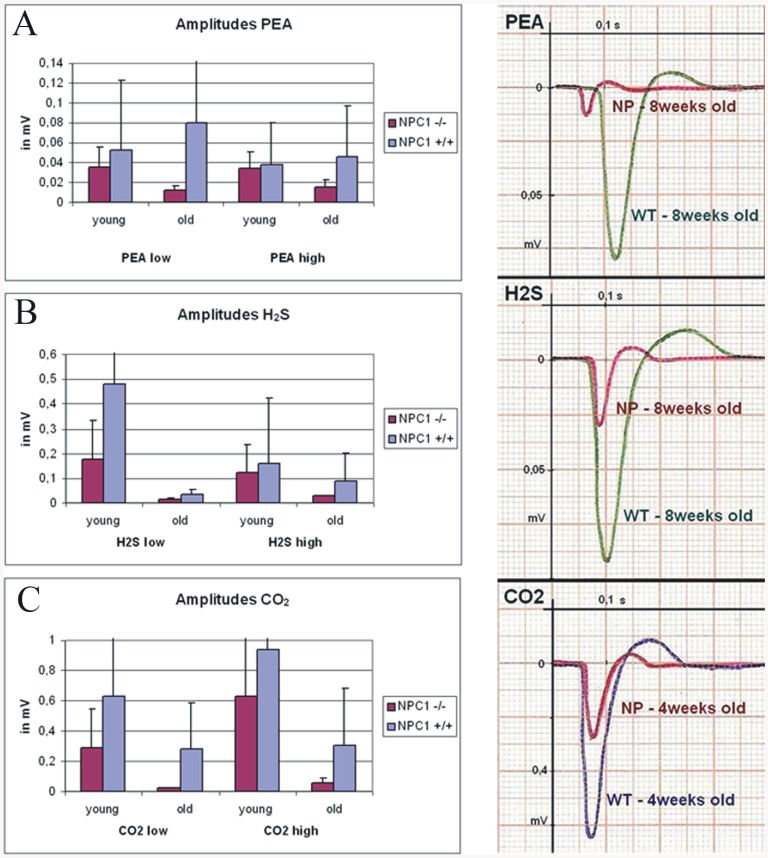
On average, recordings from the olfactory epithelium exhibited decreased amplitudes after exposure to PEA (A), H_2_S (B), and C0_2_ (C) in NPC1^−/−^ mice. Differences tended to be significant for adult (67 d) but not for young animals (31 d). Individual EOG recordings are shown on the right side. X-axis gives the time after volatile stimulation (at 0 sec) and y-axis shows the response in mV for a wild type (WT) and a NPC1^−/−^ mouse (NP). The response amplitude is the minimum of each graph and the latency is the time between the stimulus and the minimum. Duration of recordings shown −400 msec.

Electro-olfactograms of the olfactory mucosa suggest that NPC1^−/−^ animals exhibit olfactory deficits (Fig. 10). Stimuli were chosen to selectively activate the olfactory (phenyl ethyl alcohol, PEA and hydrogen sulphide, H_2_S) and trigeminal (carbon dioxide, CO_2_) nerves. Recordings of mucosa sum potentials revealed a tendency of decreased amplitudes after exposure to PEA (A), H_2_S (B), and CO_2_ (C) in NPC1^−/−^ mice. The difference between groups was more distinct in adult (67 d) than in young animals (32 d).

### Trigeminal ganglion

Taken into consideration the fact that CO_2_ stimuli activate selectively the trigeminal nerve with little or no concomitant olfactory stimulation, we further tried to clarify the basis for lower amplitude in NPC1^−/−^ mice compared to controls. As expected, electron microscopy revealed pathological changes in form of material accumulation in ganglion cells and satellite cells (Fig. 9E).

Prominent concentric lamellar structures became obvious also in the Schwann cells in the trigeminal nerve (not shown).

## Discussion

NPC1 is a recessive lipid storage disorder characterized by severe, progressive neurodegeneration. Devastating degeneration results in cognitive impairment, ataxia and death, most often in childhood. Natural history studies and therapy trials are difficult to perform in this disorder due to the relatively low incidence and the heterogeneity of disease in human patients. In recent years mutant mouse models have facilitated the understanding of structural and molecular events occurring as a result of NPC1 gene mutation. A mouse model of NPC1 disease, the BALB/cJ NPC1^NIH^, has been shown to resemble human NPC1 and is used to investigate closely molecular and biochemical aspects of the disease [13]. The NPC1 gene is mutated in these mice, and the locus belongs to the same complementation group as human NPC1.

### Olfactory dysfunction may be an early sign in neurodegenerative diseases

The present study was undertaken to examine the olfactory system at histological, ultrastructural and functional levels. While one of the earliest clinical symptoms reported by patients with neurodegenerative diseases such as Alzheimer's and Parkinson's diseases is olfactory dysfunction [25], [47], investigations of the olfactory system are scarce in lysosomal storage diseases both in humans and experimental animal models. Assessment of olfactory function in Gaucher patients revealed significantly lower scores compared with healthy individuals [29]. In NPC1 disease, the structures of olfactory epithelium and central pathways as well as olfactory function have not been studied so far.

### General symptoms of NPC1^−/−^ mutant mice

NPC1^−/−^ mutant mice are asymptomatic and even undistinguishable from their littermates at birth. The earliest definitive symptoms of the disease become apparent by 4 to 6 weeks of age, and as they reach adulthood symptoms of ataxia and hind limb paralysis emerge [48]. Abnormalities in intracellular cholesterol transport with subsequent accumulation of lipids could be found in many organs of these mutant mice, including brain [49]. At the cellular level, NPC1^−/−^ mice show an age-related loss of neurons in the prefrontal cortex, thalamus, brainstem, and of cerebellar Purkinje cells, as well as activation of microglia and astrocytes with phenotypes that are similar to those observed in human NPC1 disease [16], [48], [50].

### Neurodegeneration, activation of peripheral macrophages and cerebral glial cells in components of the olfactory system

As expected, the main findings of the present study reveal pronounced peripheral and central neurodegeneration as well as glia activation in the olfactory system- a part of CNS which has not been examined in NPC1 before. The neurodegeneration in NPC1 has been demonstrated to be an autonomous process, caused primarily by the lack of NPC1 in the central nervous system [51]. Prior mammalian studies have revealed that NPC1 predominantly localized in glia [52], and both, astrocytes and microglia have been suggested to mediate inflammation and neurodegeneration in NPC1 mice [53], [54]. It has been shown that at two weeks of age the reactive astrocytes were only observed in the ventral lateral thalamus, while another two weeks later massive astrogliosis was seen throughout the entire brain of NPC1^−/−^ mice [22]. The astroglial reaction coincided with up-regulation of the cytokine, interleukin-1beta, in most, but not all brain regions. It has been previously suggested that proinflammatory signals that trigger glial inflammatory responses originate from astrocytes as a consequence of NPC1 loss in these cells [55].

Although glial cells have been proposed to be the major target for neuropathology in NPC1 [56], there has been increasing evidence in recent years that neuronal death is the predominant factor, which causes glia activation. For example, in the cerebellum, gliosis was not seen in areas where Purkinje cells were still present, and the only concentration of astrocytes was seen in or near sites of Purkinje cells loss [57]. These authors suggested that glial cell activity remains responsive to neurons and does not occur solely because of NPC1 loss in glia. However, the situation in the olfactory system seems to be relatively complex and the question whether neuronal death mechanistically causes glial activation or vice versa cannot be answered univocally. On the one hand, we see pronounced neuronal death in the periphery (OE), while, on the other hand, massive glia activation is observed in the interface between axons of incoming ORN and mitral cells/periglomerular cells in the OB without distinctive neuronal loss (for example, in the granular layer of OB).

Up-regulation of Gal-3, CatD and GFAP in the cortical layers of the OB underlines the critical role and location of the OB as a possible entrance gate for noxious substances from the periphery. Remarkably, the OB is the site of most intense astroglia and microglia activation in the whole brain in NPC1^−/−^. This may reflect the fact that ORN and their ensheathing cells enter the OB without a distinct barrier between olfactory mucosa and brain tissue [58], [59]. The unhindered passage of peripheral olfactory structures has been discussed as a key factor for viral invasion of the OB [60] and even in the pathogenesis of Parkinson's disease (dual hit hypothesis) [61].

Astrocytes are known to be the major site of cholesterol synthesis that is required during brain development and repair. The endogenously produced cholesterol is secreted by astrocytes via the transporter ABCA-1 [62]. Thus, the overall observed elevation of the protein ABCA-1 in our study can be explained by increased number of astrocytes.

Another significant finding of our study was the increased expression of the lysosomal enzyme CatD in OE and OB. CatD along with Gal-3 expression can be related to invaded macrophages and increased lysosomal activity. Increased levels and activity of CatD have been previously shown in hippocampus and cerebellum of NPC1^−/−^ mice [63]. It has been suggested that increased expression/release/activation of CatD in neurons and astrocytes can trigger neurodegeneration and development of NPC pathology [64]. Elevated levels of CatD expression and activity have been also shown to be involved in the pathogenesis of Alzheimer disease, atherosclerosis and cancer [65].

### The trigeminal system is affected in NPC pathology

In contrast to primarily telencephalic olfactory projections, fibers of the trigeminal system travel via the trigeminal ganglion to brainstem nuclei and reach postcentral gyri after relaying in the ventral posteromedial thalamic nucleus [33], [66]. Apart from mechanosensory inputs, trigeminal fibers also carry “general chemosensory” modalities [67], which allow an increasingly important crosstalk with olfactory stimuli [68]. The myelin-like deposits in trigeminal ganglion cells and satellite cells suggest impairment of trigeminal function in NPC1^−/−^ mice, as demonstrated by electrophysiology.

As a consequence of all pathological changes observed at the structural level we further demonstrate functional impairment in olfaction as shown by decreased amplitudes after exposure of the OE to different olfactory and trigeminal stimuli. Interestingly, the deterioration was more evident in adult animals, rather than in young ones. According to the literature, distinct clinical symptoms in NPC1 occur after the neuronal impairment has reached a threshold level [69]. Previously, impaired retinal function has been shown in the same mouse model [19]. Similarly to the olfactory system, also in the retina the lipid accumulation leads to destructive cellular changes, deformation of layers and degeneration of photoreceptors.

One of the important implications as a result from our observations in this murine model is that both the olfactory and trigeminal impairment are early events in NPC1 pathogenesis, at least in comparison with impairment of motor acuity that does not occur before 42–49 days of age [15], [32]. These data will lead to future studies, focusing on the olfactory system of NPC1 patients. The olfactory system offers the opportunity of in vivo functional measurements using simple psychophysical or electrophysiological tests. The latter could become a helpful tool to estimate the degree of neurodegeneration and monitor a therapy success, e.g. during a combined treatment with cyclodextrin/allopregnanolone and miglustat in follow-up studies that has become available in recent years [70], [71]. Another intriguing question to be addressed in the future is how the olfactory system may compensate for neuronal loss in NPC1. Studies concerning the behavior of migrating neuronal precursors are in progress.

## Supporting Information

Figure S1 (A) body weight of young and adult NPC1^−/−^ mice in comparison with age-matched NPC1^+/+^ group. (B) whole brain weights of NPC1^+/+^ and NPC1^−/−^ mice at P64 after perfusion. The difference between groups is significant (p = 0.0221). Data are presented as mean ± SD.
(TIF)

Figure S2 **Comparative electron micrographs of OE in young (32d) and adult (67d) NPC1^−/−^animals.** (A) Myelin-like deposits are already visible in most supporting cells (S) and ORN (O) of young animals. (B) In addition, basally located cells are more affected in adult mice. BG, excretory duct of a Bowman gland. The basal lamina is indicated with dotted lines. Scale bar: 5 µm.
(TIF)
