# Supplementary figures and images for: Olfactory Deficits in Niemann-Pick Type C1 (NPC1) Disease
Source: PLoS One. 2013 Dec 31;8(12):e82216. doi: 10.1371/journal.pone.0082216 (PMC3877006; doi:10.1371/journal.pone.0082216)

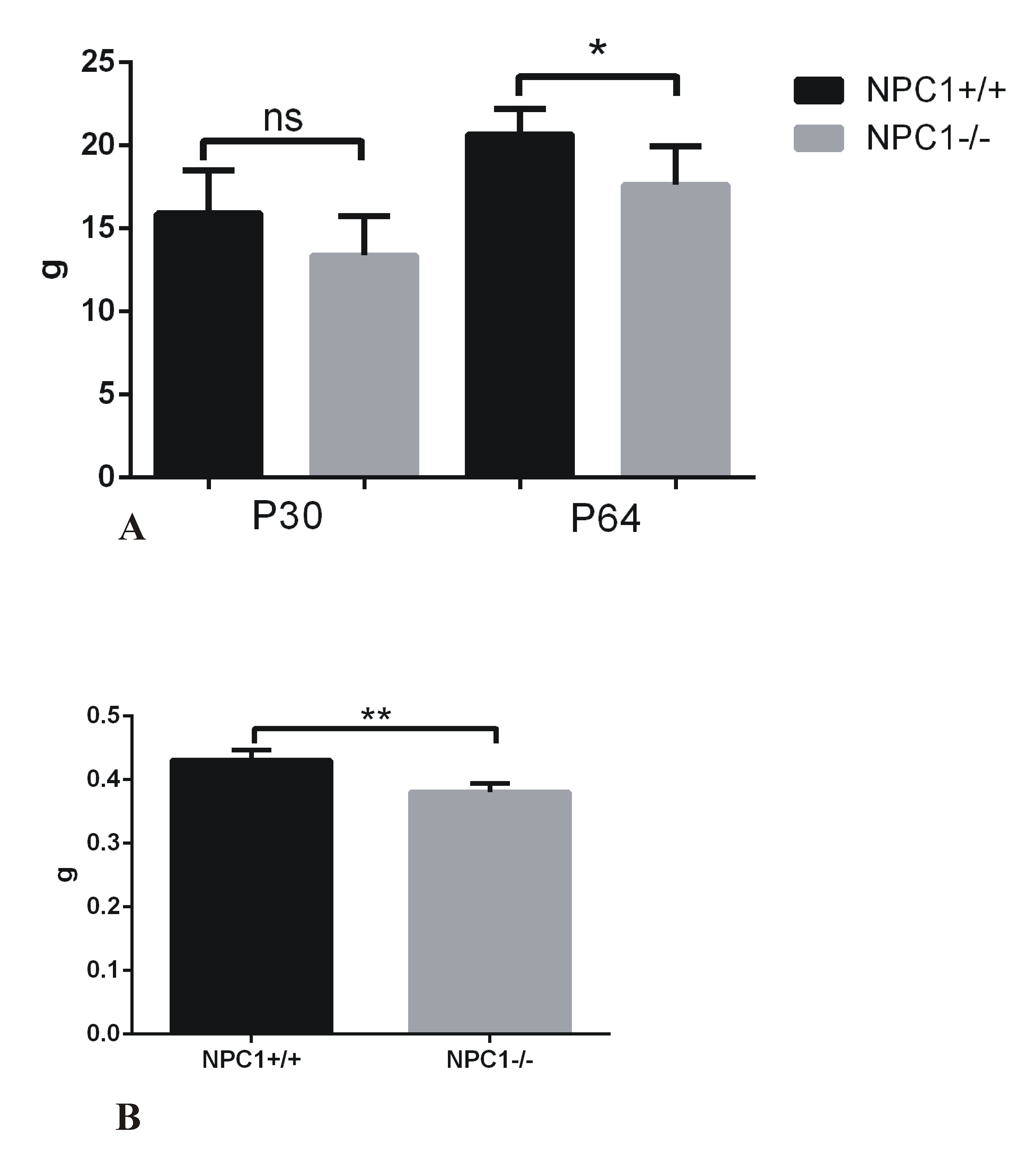

Supplement: Figure S1 — (A) body weight of young and adult NPC1−/− mice in comparison with age-matched NPC1+/+ group. (B) whole brain weights of NPC1+/+ and NPC1−/− mice at P64 after perfusion. The difference between groups is significant (p = 0.0221). Data are presented as mean ± SD. (TIF) [file pone.0082216.s001.tif]

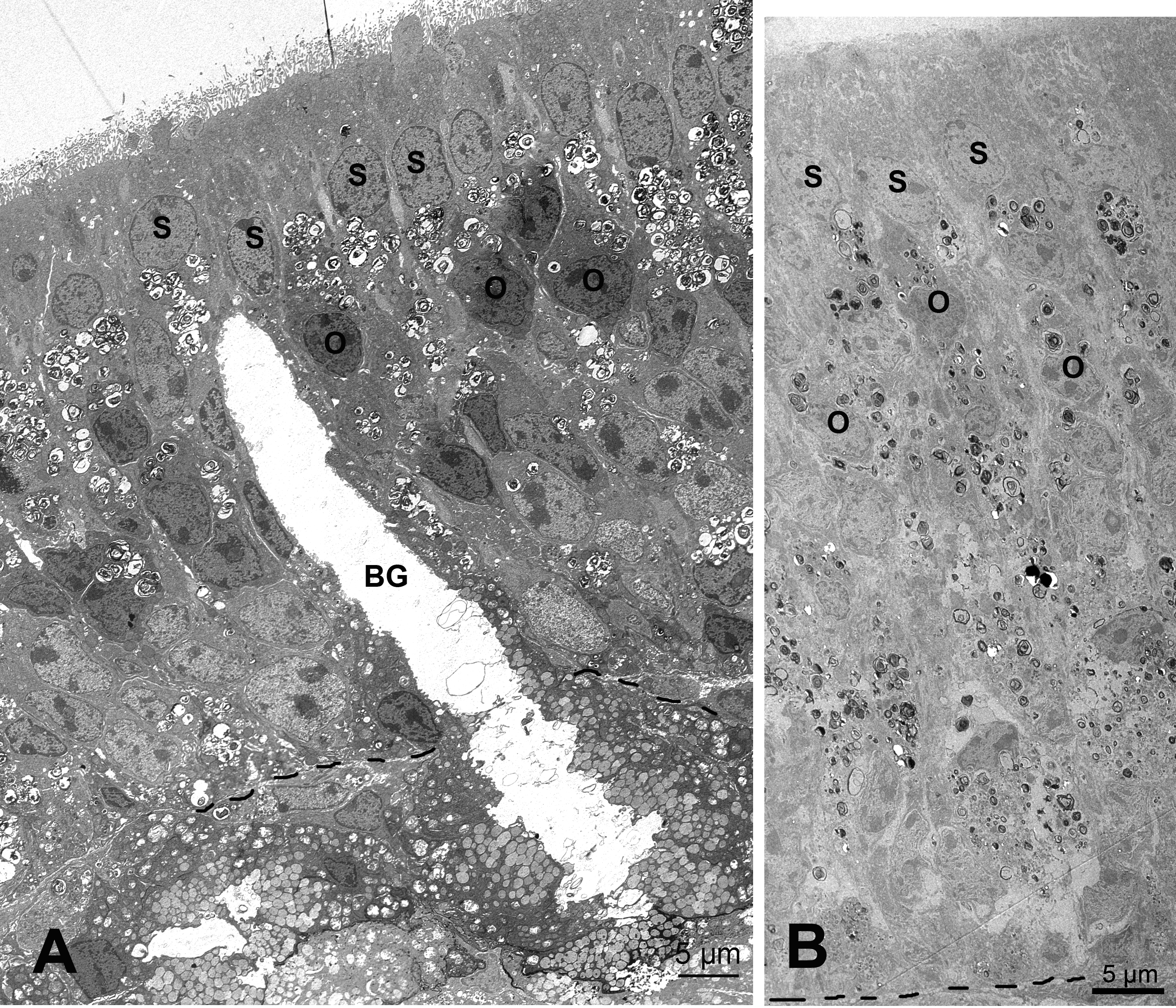

Supplement: Figure S2 — Comparative electron micrographs of OE in young (32d) and adult (67d) NPC1−/−animals. (A) Myelin-like deposits are already visible in most supporting cells (S) and ORN (O) of young animals. (B) In addition, basally located cells are more affected in adult mice. BG, excretory duct of a Bowman gland. The basal lamina is indicated with dotted lines. Scale bar: 5 µm. (TIF) [file pone.0082216.s002.tif]
